# Associations of maternal immune response with MeHg exposure at 28 weeks’ gestation in the Seychelles Child Development Study

**DOI:** 10.1111/aji.13046

**Published:** 2018-09-17

**Authors:** Emeir M. McSorley, Alison J. Yeates, Maria S. Mulhern, Edwin van Wijngaarden, Katherine Grzesik, Sally W. Thurston, Toni Spence, William Crowe, Philip W. Davidson, Grazyna Zareba, Gary J. Myers, Gene E. Watson, Conrad F. Shamlaye, J. J. Strain

**Affiliations:** ^1^ Nutrition Innovation Centre for Food and Health (NICHE) School of Biomedical Sciences University of Ulster Coleraine Northern Ireland; ^2^ School of Medicine and Dentistry University of Rochester Rochester New York; ^3^ Child Development Centre Ministry of Health Mahé Republic of Seychelles

**Keywords:** cytokines, immune function, methylmercury, n‐3 PUFA, pregnancy, Th1, Th2

## Abstract

**Problem:**

Maternal methylmercury (MeHg) exposure may be associated with immune response during pregnancy.

**Method of study:**

In the high fish‐eating Seychelles Child Development Study Nutrition Cohort 2, we examined the association between maternal MeHg, polyunsaturated fatty acids (PUFA), and immune markers (Th1:Th2; TNF‐α, IL‐1β, IFN‐γ, IL‐2, IL‐4, IL‐5, IL‐10, MCP‐1, TARC, sFlt‐1, VEGF‐D, CRP and IL‐6) at 28 weeks’ gestation. Linear regression examined associations between MeHg exposure and immune markers with and without adjustment for PUFA.

**Results:**

In all models, as MeHg concentrations increased, the Th1:Th2 ratio, total Th1 and individual Th1 (IL‐1β, IL‐2, TNF‐α) concentrations decreased. MeHg was not associated with total Th2 cytokines but was associated with a decrease in IL‐4 and IL‐10. MeHg was positively associated with TARC and VEGF‐D and negatively associated with CRP. There was a significant interaction between MeHg and the n‐6:n‐3 ratio, with MeHg associated with a larger decrease in Th1:Th2 at higher n‐6:n‐3 PUFA ratios. The n‐3 PUFA were associated with lower CRP, IL‐4 and higher IFN‐γ. The n‐6 PUFA were associated with higher IL‐1β, IL‐2, TNF‐α, IL‐4, IL‐10, CRP and IL‐6.

**Conclusion:**

Maternal MeHg was associated with markers of immune function at 28 weeks’ gestation. A significant interaction between MeHg and the n‐6:n‐3 ratio on the Th1:Th2 ratio suggests that the n‐3 PUFA may mitigate any immunosuppressive associations of MeHg. The n‐3 and n‐6 PUFA were associated with suppressive and stimulatory immune responses, respectively. Overall, the associations were of small magnitude, and further research is required to determine the clinical significance.

## 1 INTRODUCTION

The immune system during pregnancy is defined by maternal‐placental tolerance facilitated via immune cell activation and cytokine release.^1^, ^2^ In normal pregnancy, the balance of T‐helper (Th) 1 (cell‐mediated immunity) to Th2 (humoral immunity) cell activity is characterized by a more pronounced Th1 proinflammatory response in the first trimester, required for successful implantation.^2^, ^3^ Thereafter, there is a shift towards greater Th2 anti‐inflammatory cell activity and downregulation of Th1 cell activity in the second and third trimesters to allow maternal tolerance to the developing foetus.^4^, ^5^, ^6^ The immune response returns to a Th1 proinflammatory phase towards the end of the third trimester to initiate the labour process.^7^ It has been suggested that a lower index of Th1:Th2 immune response in the second trimester is supportive for successful physiologic pregnancy.^2^, ^8^ In the second trimester, Th2 cells secrete predominantly anti‐inflammatory cytokines including interleukin (IL)‐4, IL‐5 and IL‐10 which, in combination with a reduction in inflammatory Th1 cytokines IL‐1β, IL‐2 and interferon‐gamma (IFN‐γ), have been associated with healthy term pregnancy.^9^, ^10^ Therefore, specific balance between maternal proinflammatory and anti‐inflammatory cytokines throughout pregnancy is believed to be critical for the success of a pregnancy and favourable birth outcomes.^11^ The normal variation, however, in the magnitude of these immunologic changes during pregnancy has not been fully defined.

Pregnant women are encouraged to consume fish because fish are rich in nutrients including long‐chain n‐3 polyunsaturated fatty acids (PUFA), which are important for normal brain development.^12^ However, fish consumption advisories have been issued based on the limited epidemiological evidence that there might be neurotoxicity from the small amounts of naturally acquired methylmercury (MeHg) which is found in all fish. MeHg exposure has been associated with markers of immune response^13^ albeit its association with immune function in pregnancy is not known. Cytokine dysregulation, however, as a result of MeHg exposure has been reported in animal models.^14^ Evidence from in vitro and murine models suggests that MeHg reduces T‐ and B‐cell responses, and this immunosuppressive response has been demonstrated *in vivo* and is associated with changes in immune function.^15^, ^16^ Most work in this area has focused on inorganic Hg species. Treatment of human peripheral blood mononuclear cells (PBMCs) with mercury chloride (HgCl_2_) was found to significantly increase the release of the proinflammatory cytokines tumour necrosis factor (TNF)‐α, IL‐1β and IL‐6 in a dose‐dependent manner^17^, ^18^, ^19^ and to reduce the concentration of the anti‐inflammatory markers IL‐1Ra and IL‐10.^18^ In contrast, MeHg exposure is thought to produce an initial immunosuppressive response, characterized by reduced B‐ and T‐cell numbers followed by a delayed Th1 response, which may persist long after exposure.^13^, ^16^ One observational study of high fish‐eating mothers found an elevated IgG response apparent with MeHg exposure and may be indicative of an autoimmune type response.^20^ No other study, to our knowledge, has investigated MeHg exposure on maternal immune response.

The n‐3 PUFA have been suggested to protect against oxidative insult and to have beneficial effects on immune function and inflammation in pregnancy.^21^, ^22^, ^23^ Long‐chain n‐3 PUFA, together with long‐chain n‐6 PUFA (eg arachidonic acid [AA]), have important roles in inflammation by acting as precursors for eicosanoid and docosanoid signalling molecules. These signalling molecules, including the proresolvins and lipoxins, have roles not only in inflammation but also in inducing active resolution of inflammation and thereby having a protective function against uncontrolled inflammation.^24^, ^25^ Inflammation is nonspecific, and although regulated by cytokine expression, it does not always follow the Th1:Th2 hypothesis.^26^ Therefore, the beneficial effects of n‐3 PUFA may not be reflected through favouring either a Th1 or Th2 response.^27^ However, the anti‐inflammatory actions of n‐3 PUFA are widely acknowledged and have largely been reported to involve the suppression of proinflammatory cytokines and the enhanced production of more anti‐inflammatory cytokines, possibly at the eicosanoid rather than Th‐cell level.^28^, ^29^, ^30^, ^31^ The n‐6 PUFA‐derived eicosanoids are generally proinflammatory,^32^ while the anti‐inflammatory effects of n‐3 PUFA (suppression of Th1 and enhancement of anti‐inflammatory Th2 cytokines) are widely reported.^28^ Therefore, a higher concentration of n‐3 PUFA along with a lower n‐6:n‐3 ratio is suggested to be important in regulating inflammation. Previously in the Seychelles Child Development Study (SCDS) Nutrition Cohort 2 (NC2), we reported that MeHg‐induced inflammation might depend on the physiologic balance of n‐6 to n‐3 PUFA.^33^ Therefore, we examined the associations between maternal MeHg exposure, PUFA and maternal blood markers of inflammation at 28 weeks’ gestation in the NC2 to better understand the risk/benefit of fish consumption during pregnancy.

## 2 METHODS

### 2.1 Study population

Participants for this study are part of the SCDS Nutrition Cohort 2 (NC2) which has been described in detail elsewhere.^33^ In brief, between 2008 and 2011, a large mother‐child cohort (n = 1518 eligible mothers) was recruited. Mothers were enrolled during their first antenatal visit (from 14 week of gestation) at eight health centres across the island of Mahé. Inclusion criteria for NC2 included being native Seychellois, being =>16 years of age, and having a singleton pregnancy, with no obvious health concerns. The study was reviewed and approved by the Seychelles Ethics Board and the Research Subjects Review Board at the University of Rochester.

### 2.2 Blood collection

At 28 weeks of gestation, nonfasting maternal blood samples were collected by antecubital venepuncture into evacuated serum and EDTA tubes and were immediately processed by centrifuging at 700 *g* for 15 minutes. All samples were stored at −80°C and maintained at this temperature throughout their shipment to Ulster University for storage and analysis.

### 2.3 MeHg exposure

Maternal whole‐blood samples were shipped to the University of Rochester for total Hg (THg) analysis using a PSA Millennium Merlin System (PS Analytical, Kent, UK). Our limit of detection for THg in blood is ~0.01 ng/mL, depending on sample volume^34^. Standard Reference Materials (SRM 966 Toxic Metals in Bovine Blood and Seronorm 201605) were purchased from the National Institute of Standards and Technology (Gaithersburg, MD) and SERO (Billingstad, Norway), respectively. We did not speciate Hg. All Hg results presented as MeHg are total Hg (THg) based on the assumption that ~80% of THg in whole blood is MeHg within the Seychelles population.^35^, ^36^

### 2.4 Inflammatory markers

Serum samples were analysed at Ulster for a panel of inflammatory markers by Meso Scale Discovery (MSD) multiplex assay (Meso Scale Diagnostics, LLC.). These assays yield greater sensitivity and larger dynamic range and require less sample volume than traditional methods for immunoassays.^37^ In preliminary analysis, we screened 50 maternal samples using a V‐PLEX human biomarker 40‐Plex MSD panel of immune markers. Based on significant associations in our preliminary analysis, reported MeHg associations in the literature, and reported n‐3 long‐chain polyunsaturated fatty acid status, we determined a panel of inflammatory markers to be assessed in maternal serum samples.^20^ Cytokines that were measured for cell‐mediated inflammatory reactions (Th1) included IL‐1β, IL‐2, IFN‐γ and TNF‐α. The cytokines associated with Th2‐cell activity included IL‐4, IL‐5 and IL‐10. In addition, we measured IL‐6 which plays a dual role in Th1:Th2 differentiation^38^ and is largely a proinflammatory cytokine which has been shown to be stimulated in vitro following MeHg exposure.^39^, ^40^ We measured two pregnancy‐associated chemokines, monocyte chemotactic protein‐1 (MCP‐1) and thymus‐ and activation‐regulated chemokine (TARC) and two angiogenesis markers noted to be important for maintenance of placental vascular development and blood flow, soluble fms‐like tyrosine kinase‐1 (sFlt‐1) and a member of the vascular endothelial growth factor (VEGF) family, VEGF‐D. Flow cytometry analysis was not possible given the retrospective nature of this study, and therefore, the sum of cytokines concentrations (pg/mL) was used to reflect the Th1:Th2 ratio as follows: Th1‐type cell activity: IL‐1β, IL‐2, IFN‐γ and TNF‐α; and Th2‐cell activity: IL‐4, IL‐5 and IL‐10. We analysed the remaining markers individually: CRP, IL‐6, MCP‐1, TARC, sFlt‐1 and VEGF‐D. CRP (mg/L) was measured by Ultra‐Sensitive diagnostic kit using the iLab 650 Clinical Chemistry Analyzer.

### 2.5 PUFA status

Maternal serum samples were used to determine total PUFA analysis at Ulster University, using a method adapted from that of Folch et al^41^ Fatty acid methyl esters were detected and quantified by using the gold‐standard technique of gas chromatography‐mass spectrometry (7890A‐5975C; Agilent) using heptadecanoic acid (C17:0) as the internal standard, as previously described.^42^ All analytic standards were of =>99% purity and purchased from Sigma‐Aldrich. Total serum PUFA status was chosen as a biomarker of PUFA concentrations representing the triacylglycerol fraction, to which the majority of circulating PUFA are bound during pregnancy.^43^ We measured the individual PUFA (linoleic acid [LA], arachidonic acid [AA], α‐linolenic acid [ALA], eicosapentaenoic acid [EPA] and docosahexaenoic acid [DHA]), and results were presented as mg/mL to indicate physiologic quantities.

### 2.6 Covariates

Our models adjusted for variables known to impact immune response as covariates in the regression models: child sex,^44^ maternal age,^45^ maternal smoking status,^46^ maternal BMI^47^ and gestational age.^48^ Child sex was recorded at birth. Mothers reported their age and smoking status at enrolment to the study using questionnaires administered by trained nurses. When infants were ~20 months of age, maternal height and weight were recorded, from which their postnatal BMI was calculated (BMI = weight (kg)/height (m)^2^). Data on prepregnancy BMI were unavailable, and we adjusted for postnatal BMI as data from our NC1 showed it to strongly correlate (r = 0.93) with maternal BMI prepregnancy. Gestational age (weeks) at blood collection was calculated using the gestational age at delivery and date of blood collection.

### 2.7 Statistical analysis

From our final database of mothers (n = 1473) with complete inflammatory marker data, we removed two mothers with extremely high concentrations of multiple inflammatory markers indicating possible illness and two additional mothers with a gestational age at blood collection (18 and 40 weeks) very far from the average of 28 weeks. Undetectable inflammatory marker data are common in multiplex assays; however, the MSD platform is extremely sensitive, and as such, ≥80% of concentrations were above the LLOD for most markers. For undetectable values, we inputted LLOD/√2 values as a replacement value.^49^ The lowest rates of detection were observed for IL‐2 and IL‐4 where only 36% of all samples were above the LLOD. Excluding these markers from Th1, Th2 and Th1:Th2 variables did not affect the strength or significance of the modelled relationships with PUFA or MeHg. Therefore, we included these markers both as individual variables and within the Th1 and Th2 summed variables.

We fit a series of linear regression models to determine the association of immune markers with MeHg exposure in pregnancy, and the potential influence of PUFA on this relationship. Model assumptions, including linearity, constant variance, normality of residuals and presence of outlying influential observations, were checked graphically using standard methods.^50^ To better satisfy regression assumptions, we used the natural logarithmic transformation of all inflammatory markers in the models. Because of the presence of zeros in some markers, we added a constant of +1 to each individual inflammatory marker data, and to the total Th1 and Th2 prior to the natural log transformation. After excluding subjects with missing values for model covariates (including 119 missing MeHg and 50 missing PUFA), a total of n = 1158 mothers had complete data for inclusion in the final regression models with n = 1156 for models examining CRP owing to two additional subjects having no CRP data. All results presented are based on n = 1158 (or n = 1156 for CRP). For each outcome, we fit four models, where each model adjusted for gestational age at blood collection, maternal age, smoking status, maternal BMI and child sex. The four models additionally adjusted for the following: (Model 1) MeHg; (Model 2) MeHg, n‐3 PUFA and n‐6 PUFA; (Model 3) MeHg and the n‐6:n‐3 PUFA ratio; and (Model 4, interaction model) MeHg, tertiles of the n‐6:n‐3 ratio (low/medium/high), and the interactions of MeHg and n6:n3 ratio tertiles as previously reported.^33^

## 3 RESULTS

Table 1 displays the demographic characteristics of the mothers. The mean (SD) age of the cohort was 27.17 ± 6.27 years, and the mean (SD) gestational age of blood collection was 27.89 ± 1.14 weeks. Mean (SD) whole‐blood MeHg was 18.14 ± 10.97 μg/L, and the PUFA n‐6:n‐3 ratio was 4.34 ± 1.61. Concentrations of inflammatory markers (untransformed) and their detection rates are outlined in Table 2. The average (SD) Th1:Th2 cytokine ratio was observed to be 7.88 ± 10.75.

**Table 1:** Maternal characteristics including MeHg and PUFA concentrations (n = 1158)

	N (%)	Mean (SD)	25th, 75th percentile
Blood gestational age (wk)		27.89 (1.14)	27.43, 28.43
Maternal age (y)		27.17 (6.27)	22.19, 31.48
Smoking (Y)	10 (1)		
Maternal BMI (kg/m^2^)		26.99 (6.55)	21.83, 31.04
Child sex (F)	548 (47)		
Blood MeHg (ppb)		18.14 (10.97)	10.70, 22.67
Serum n‐3 PUFA (mg/mL)		0.27 (0.09)	0.20, 0.33
Serum n‐6 PUFA (mg/mL)		1.10 (0.29)	0.89, 1.29
n‐6:n‐3 PUFA		4.34 (1.61)	3.25, 4.98

BMI, body mass index; F, female; MeHg, methylmercury

**Table 2:** Maternal serum inflammatory marker concentrations (n = 1158)

	Mean (SD)	25th, 75th percentile	% Detected (>LLOD)	LLOD
Th1				
IL‐1β	0.32 (0.62)	0.03, 0.39	82.6	0.04
IL‐2	0.29 (0.60)	0.06, 0.32	36.1	0.09
IFN‐γ	5.63 (20.26)	1.03, 4.91	84.4	0.2
TNF‐α	7.26 (7.55)	3.64, 8.89	100	0.04
Th2				
IL‐4	0.15 (0.58)	0.01, 0.06	35.5	0.02
IL‐5	1.36 (2.22)	0.40, 1.50	100	0.22
IL‐10	1.59 (7.85)	0.27, 1.70	90.6	0.03
Additional markers				
IL‐6	0.96 (1.44)	0.23, 1.12	88.7	0.06
MCP‐1	76.06 (94.02)	43.63, 87.46	100	0.09
TARC	99.39 (124.16)	47.56, 121.88	100	0.22
sFlt‐1	2160.19 (1786.59)	1221.93, 2589.82	100	0.56
VEGF‐D	743.88 (374.52)	492.37, 913.98	100	2.53
CRP	3.52 (2.70)	1.38, 4.84	98.9	1
Th1	13.50 (22.56)	5.80, 14.83		
Th2	3.10 (8.23)	1.12, 3.36		
Th1:Th2	7.88 (10.75)	3.01, 8.47		

Data are untransformed. LLOD: lower limit of detection. Units are pg/mL except for CRP: mg/L

### 3.1 MeHg and Th1:Th2, total Th1 and total Th2

Covariate‐adjusted associations between maternal blood MeHg, PUFA and the inflammatory markers (presented as exponentiated slopes, representing multiplicative associations) from fitting Models 1‐3 are presented in Table 3. Maternal blood MeHg was associated with a statistically significant decrease in the Th1:Th2 ratio for all three main‐effects models. Each 1 μg/L increase in maternal blood MeHg was associated with a decrease in Th1:Th2 from 100% to 99.5% (*P* < 0.003), for example, a 0.5% decrease, with or without adjustment for PUFA. Maternal blood MeHg was associated with a significant decrease in Th1 for all three main‐effects models. In covariate‐adjusted models, each 1 μg/L increase in maternal blood Hg was associated with a 0.7% decrease in Th1 (*P *< 0.001); with or without adjustment for PUFA. There were no significant associations between maternal blood MeHg, n‐3 PUFA and Th2 in any of the models after adjusting for covariates. N‐6 PUFA was associated with a significant 17.9% increase in Th2 (*P *= 0.013).

**Table 3:** Main‐effects models reporting covariate‐adjusted associations between MeHg, PUFA and the markers of inflammation^a^^,^ a

Inflammatory marker	MeHg	MeHg, n‐3 PUFA and n‐6 PUFA	MeHg and n‐6:n‐3 PUFA
Th1/Th2			
MeHg	**0.995 (0.003)**	**0.995 (0.004)**	**0.995 (0.003)**
n‐3 PUFA		1.354 (0.210)	
n‐6 PUFA		1.099 (0.194)	
n‐6/n‐3			0.990 (0.417)
Total Th1			
MeHg	**0.993 (<0.001)**	**0.993 (<0.001)**	**0.993 (<0.001)**
n‐3 PUFA		1.439 (0.137)	
n‐6 PUFA		**1.295 (<0.001)**	
n‐6/n‐3			1.004 (0.737)
IFN‐γ			
MeHg	0.997 (0.258)	0.997 (0.214)	0.997 (0.27)
n‐3 PUFA		**2.089 (0.020)**	
n‐6 PUFA		1.196 (0.060)	
n‐6/n‐3			0.994 (0.71)
IL‐1β			
MeHg	**0.998 (0.002)**	**0.998 (0.007)**	**0.998 (0.006)**
n‐3 PUFA		0.843 (0.070)	
n‐6 PUFA		**1.105 (<0.001)**	
n‐6/n‐3			**1.015 (0.001)**
IL‐2			
MeHg	**0.998 (0.015)**	**0.998 (0.031)**	**0.998 (0.031)**
n‐3 PUFA		0.9023 (0.312)	
n‐6 PUFA		**1.105 (0.001)**	
n‐6/n‐3			**1.013 (0.010)**
TNF‐α			
MeHg	**0.992 (<0.001)**	**0.993 (<0.001)**	**0.993 (<0.001)**
n‐3 PUFA		0.938 (0.757)	
n‐6 PUFA		**1.28 (<0.001)**	
n‐6/n‐3			1.012 (0.231)
Total Th2			
MeHg	0.998 (0.164)	0.998 (0.197)	0.998 (0.216)
n‐3 PUFA		1.063 (0.781)	
n‐6 PUFA		**1.179 (0.013)**	
n‐6/n‐3			1.014 (0.206)
IL‐4			
MeHg	**0.999 (0.041)**	0.999 (0.095)	0.999 (0.088)
n‐3 PUFA		**0.823 (0.019)**	
n‐6 PUFA		**1.066 (0.011)**	
n‐6/n‐3			**1.014 (0.001)**
IL‐5			
MeHg	1.001 (0.437)	1.001 (0.533)	1.001 (0.456)
n‐3 PUFA		1.273 (0.192)	
n‐6 PUFA		1.043 (0.446)	
n‐6/n‐3			1.001 (0.904)
IL‐10			
MeHg	**0.996 (0.005)**	**0.996 (0.010)**	**0.996 (0.009)**
n‐3 PUFA		0.939 (0.752)	
n‐6 PUFA		**1.179 (0.006)**	
n‐6/n‐3			1.013 (0.183)
IL‐6			
MeHg	0.998 (0.097)	0.998 (0.183)	0.998 (0.167)
n‐3 PUFA		0.88 (0.442)	
n‐6 PUFA		**1.14 (0.009)**	
n‐6/n‐3			1.012 (0.157)
MCP‐1			
MeHg	1.003 (0.191)	1.003 (0.183)	1.003 (0.178)
n‐3 PUFA		1.275 (0.483)	
n‐6 PUFA		1.175 (0.122)	
n‐6/n‐3			1.000 (0.995)
TARC			
MeHg	**1.008 (0.004)**	**1.009 (0.003)**	**1.009 (0.003)**
n‐3 PUFA		0.833 (0.635)	
n‐6 PUFA		1.019 (0.874)	
n‐6/n‐3			1.015 (0.445)
sFlt‐1			
MeHg	1.000 (0.743)	0.999 (0.732)	0.999 (0.742)
n‐3 PUFA		1.209 (0.359)	
n‐6 PUFA		1.021 (0.739)	
n‐6/n‐3			0.993 (0.473)
VEGF‐D			
MeHg	**1.004 (0.002)**	**1.004 (0.001)**	**1.004 (0.001)**
n‐3 PUFA		0.890 (0.510)	
n‐6 PUFA		1.091 (0.100)	
n‐6/n‐3			1.017 (0.052)
CRP			
MeHg	**0.996 (0.006)**	**0.997 (0.028)**	**0.996 (0.017)**
n‐3 PUFA		**0.449 (<0.001)**	
n‐6 PUFA		**1.136 (0.044)**	
n‐6/n‐3			**1.034 (0.001)**

Values shown are exponentiated slopes, representing multiplicative associations (*P*‐values; significant associations in bold).

Main‐effects models also adjusted for the following: (Model 1) MeHg; (Model 2) MeHg, n‐3 PUFA and n‐6 PUFA; and (Model 3) MeHg and n‐6:n‐3 PUFA.

a All models adjusted for gestational age at blood collection, maternal age, smoking status, maternal BMI and child sex.

**Figure 1:**
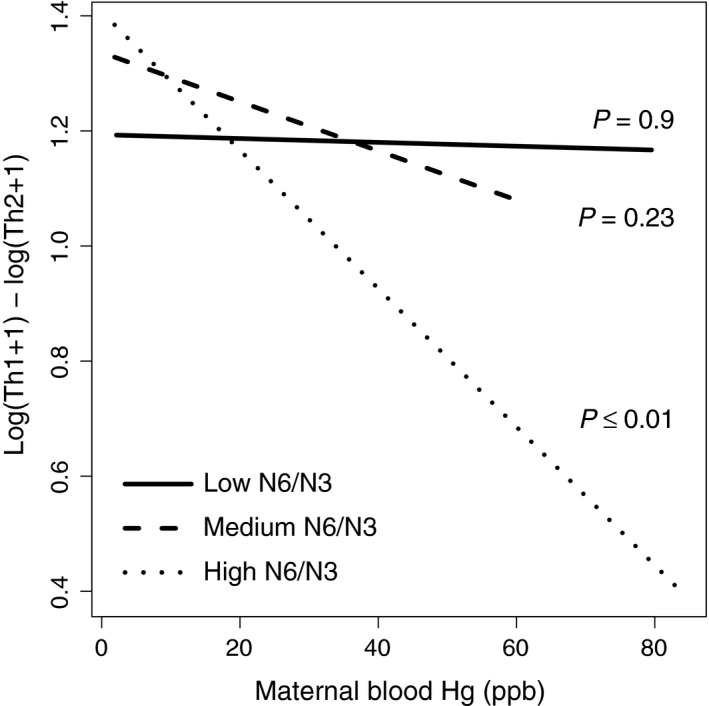
Interaction models for maternal blood MeHg exposure against Th1:Th2 cytokine ratio at 28 weeks’ gestation. Models shown are adjusted for maternal gestational age at blood collection, maternal age, smoking status, maternal BMI and child sex

In the interaction model, the association between MeHg and Th1:Th2 differed by n‐6:n‐3 tertiles (*P* = 0.02, 2‐df test; Figure 1). MeHg was associated with a decrease in Th1:Th2 in all n‐6:n‐3 tertiles, but the association was only significantly different from zero in the highest n‐6:n‐3 tertile. For those with a high n‐6:n‐3 ratio, each 1 μg/L increase in maternal blood MeHg was associated with a decrease of 1.2% in Th1:Th2 (*P *= 0.005). The interactions between MeHg and tertiles of the n‐6:n‐3 ratio were not significant for total Th1 nor total Th2.

### 3.2 MeHg and individual inflammatory markers

As outlined in Table 3, maternal blood MeHg was significantly associated with lower concentrations of IL‐1β, IL‐2, TNF‐α, IL‐10 and CRP with or without adjustment for PUFA. Maternal blood MeHg was significantly associated with lower concentrations in IL‐4 only in the model not adjusted for PUFA. Maternal blood MeHg was positively associated with TARC and VEGF‐D.

The n‐3 PUFA was associated with a significant increase in IFN‐γ and a significant decrease in IL‐4 and CRP. The n‐6 PUFA was associated with a significant increase in total Th1, total Th2, IL‐1β, IL‐2, TNF‐α, IL‐4, IL‐10, IL‐6 and CRP. The n‐6:n‐3 ratio was associated with a significant increase in IL‐1β, IL‐2, IL‐4 and CRP. The interactions between MeHg and the tertiles of the n‐6:n‐3 ratio were not significant for any of these additional markers.

### 3.3 Covariates

Maternal BMI was a significant predictor of many inflammatory markers. After adjusting for other model covariates, BMI had a positive association with IL‐1β, TNF‐α, CRP and IL‐6, and a negative association with sFlt‐1 and VEGF‐D. Maternal age was also a significant predictor of several inflammatory markers. After adjusting for covariates, maternal age had a positive association with CRP and VEGF‐D, and a negative association with sFlt‐1 (results not shown).

## 4 DISCUSSION

This is one of the first studies to examine associations between maternal MeHg exposure and immune responses in pregnancy, in a large human population, and accounting for PUFA status. At 28 weeks’ gestation, we observed that increasing maternal MeHg levels were associated with a small magnitude decrease in the Th1:Th2 ratio along with a decrease in individual and total Th1 cytokines. There was no association between MeHg and total Th2 cytokines. Our data also suggest an interaction between MeHg exposure and the balance of n‐6 to n‐3 PUFA status in relation to these immune marker associations, with MeHg being associated with a decrease in Th1:Th2 but only at higher n‐6:n‐3 PUFA ratios. This association was not apparent with the lower ratios of n‐6:n‐3; therefore, it is plausible that the n‐6 PUFA are having stimulatory actions on Th1 while a higher concentration of n‐3 PUFA may be important to mitigate this association.

An appropriate balance between maternal Th1 and Th2 cytokines is believed to be important for the continuous normal development of pregnancy and for optimal foetal outcomes. In this study, we observed an immunosuppressive association of MeHg exposure at 28 weeks’ gestation with the Th1 cytokines IL‐1β, IL‐2 and TNF‐α. This association, which was small in magnitude, was not modified by PUFA status. A recent meta‐analysis identified an association of anti‐TNF‐α therapies with an increased risk of preterm birth, spontaneous abortion and low birthweight.^51^ Any increase in TNF‐α is normally balanced with simultaneous synthesis of IL‐10, which regulates inflammation. The balance of TNF‐α and IL‐10 is proposed to be critical for pregnancy outcome with an increase in the TNF‐α:IL‐10 ratio related to early and late pregnancy complications.^52^ We found MeHg was negatively associated with both TNF‐ α and IL‐10 in this cohort, and this may affect this ratio and warrants further investigation. IL‐1β is involved in implantation and preimplantation embryo development^53^ with elevated first trimester IL‐1β associated with preterm preeclampsia.^54^ Contrary to our findings, IL‐1β and TNF‐α were previously shown to be positively associated with MeHg in a mother‐child cohort^20^ albeit measurements were taken at delivery, a time in pregnancy when proinflammatory markers would be increased by labour. IL‐2 functions in immune regulation by activating T cells specifically memory and regulatory T cells and plays a role in immune tolerance.^55^ In particular, during pregnancy a low T regulatory (Treg) cell frequency is modulated by changes in cytokine patterns and is associated with adverse pregnancy outcome.^56^ Taken together, the associations we found between MeHg and IL‐2, TNF‐α and IL‐10 warrant further investigation to determine whether MeHg is suppressing immune regulatory cytokines and to determine whether the association, which is small in magnitude, has any clinical relevance.

A lower Th1:Th2 immune response in the second trimester compared to the first trimester is supportive for physiologic pregnancy.^57^, ^58^ The Th1:Th2 ratio observed in this study is in line with what others have reported in pregnancy,^57^ and considering this cohort consisted of only healthy term pregnancies, this is to be expected. It would also suggest that the association of MeHg with the Th1:Th2 is negligible. Nevertheless, in this cohort, the Th1:Th2 ratio during pregnancy was associated with exposure to MeHg; the ratio decreased as the MeHg exposure increased. The clinical relevance of this relationship is difficult to ascertain since the normal variations in immune markers during pregnancy are still being determined. In the second and early part of the third trimester of a normal pregnancy, the production of the proinflammatory Th1 cytokines IL‐2, TNF‐α and INF‐γ is normally suppressed, whereas the production of Th2 regulatory cytokines IL‐4 and IL‐10 is enhanced, supporting central immune tolerance at the materno‐foetal interface.^59^ This change is considered important for a healthy term pregnancy.^9^, ^10^ Although we did not see any overall association with Th2, we did observe lower concentrations of IL‐4 and IL‐10 with increasing concentrations of MeHg at 28 weeks’ gestation. In pregnancy, IL‐10 is an important immunoregulatory cytokine produced by cytotrophoblasts at the materno‐foetal interface acting to maintain the balance between humoral and inflammatory responses.^60^ Low concentrations of IL‐10 in the second trimester have been linked to recurrent spontaneous abortion.^61^ Similarly, low concentrations of IL‐4 and IL‐10 have been observed in the decidua from women suffering from unexplained recurrent abortions and where spontaneous abortion has occurred during the first trimester of pregnancy.^62^ Data on the association of Hg, in particular MeHg, with IL‐10 are limited and more research is required to ascertain any immunomodulatory effects of MeHg on IL‐10 and IL‐4 at critical periods of development during pregnancy.

In this high fish‐eating cohort, adjustment for PUFA did not alter the associations between MeHg and the inflammatory markers with the exception of IL‐4, which was no longer significant after controlling for PUFA status. We observed, however, associations between both n‐3 and n‐6 PUFA with a number of the quantified immune markers, which suggest that PUFA is directly associated with the immune response at this stage of pregnancy. We observed that n‐6 PUFA appeared to enhance both the Th1 and the Th2 response and a higher n‐6:n‐3 ratio was observed to be associated with higher concentrations of IL‐1β, IL‐2, IL‐4 and CRP, supporting a more proinflammatory response. In contrast, n‐3 PUFA, as expected, was associated with lower concentrations of IL‐4 and CRP suggesting a regulatory role of n‐3 PUFA at this stage of pregnancy. This finding confirms previous results from our cohort showing an inverse relationship between n‐3 PUFA and the proinflammatory marker CRP^63^ and supports the anti‐inflammatory role of n‐3 PUFA that is widely reported.^64^

There may be an optimal balance of n‐6:n‐3 PUFA for IL‐4, as suggested by our finding that n‐6 PUFA was associated with higher and n‐3 PUFA associated with lower concentrations of this cytokine. IL‐4 is detectable at the maternal‐foetal interface throughout pregnancy having a role in placental formation, modulation of trophoblast invasion and differentiation, inducing placental angiogenesis and inhibiting proinflammatory cytokines. Furthermore, an interaction between MeHg exposure and the balance of n‐6 to n‐3 PUFA status in relation to the immune response was apparent, with MeHg being associated with a decrease in Th1:Th2 but only at higher n‐6:n‐3 PUFA ratios. Given that the association was not significant at lower ratios of n‐6:n‐3 PUFA, it may be that the n‐3 PUFA are having a regulatory effect on the MeHg immunosuppression of Th1 cytokines. Achieving an optimal balance between n‐6:n‐3 PUFA during pregnancy in controlling inflammatory responses via cytokine signalling may therefore be important for a successful pregnancy. Given the opposing associations of MeHg and n‐6 PUFA on Th1 markers, it may be plausible that n‐6 PUFA also play an important role in stimulating immune responses during pregnancy.

The n‐3 PUFA were associated with higher concentrations of IFN‐γ, a cytokine important in innate immunity and in pregnancy where in the first trimester it enhances blood flow to the implantation site and aids placental development. MeHg in this study was not associated with IFN‐γ or IL‐6 which is contrary to what others have previously reported.^65^ As pregnancy progresses, concentrations of IFN‐γ have been shown to decline reflecting Th1 cell–mediated immunity suppression.^66^ The association between n‐3 PUFA and IFN‐γ observed in this study is similar to that reported elsewhere.^67^, ^68^ The prostaglandin E2 (PGE2) decreases the production IFN‐γ; therefore, increasing n‐3 PUFA concentrations will decrease the PGE2 resulting in higher IFN‐γ concentrations.^68^ IFN‐γ is important in the response to and clearance of viral infection upregulating transcription of genes involved in cell cycle regulation, apoptosis and antigen processing/presentation; therefore, n‐3 PUFA may be having a regulatory influence via IFN‐γ.

To investigate in greater depth the potential immunomodulatory mechanisms of MeHg and potential regulation of inflammation by PUFA, we characterized additional chemokines and angiogenesis markers within this cohort. We measured TARC, a chemokine important in Th2‐cell recruitment in pregnancy. The positive association observed between maternal MeHg and TARC is interesting given that we did not see a similar positive association between MeHg and Th2 cytokines. One possible explanation for this is that elevated concentrations of TARC in the maternal circulation act to recruit Th2 cells predominantly at the materno‐foetal interface.^69^ Furthermore, most evidence suggests that TARC plays a role in the accumulation of Th2 cells in the decidua and uterus in early pregnancy.^69^ However, given that a Th2 immune response is reported to be advantageous throughout the second and early third trimester for successful pregnancy and for foetal growth, the positive association between MeHg and TARC at 28 weeks’ gestation may be seen to be beneficial and warrants further investigation. MeHg exposure was also positively associated with concentrations of VEGF‐D, a signalling protein which promotes angiogenesis. Serum concentrations of VEGF have been found to be higher during early pregnancy than in nonpregnancy,^70^ reflecting the increase in maternal blood volume and development of the utero‐placental circulation.^71^ An imbalance in angiogenesis markers has been implicated in preeclampsia,^72^ although no differences in VEGF‐D have been found between preeclamptic and normotensive pregnant women.^73^ Treatment of mast cells with inorganic HgCl_2_ was shown to stimulate VEGF‐D and IL‐6 release, which could in turn promote inflammation at the blood‐brain barrier.^19^ Further research is required to examine whether the positive association between MeHg and VEGF‐D at 28 weeks’ gestation could have implications for foetal development through potential effects on the maternal‐foetal circulation. In contrast to the Th1 and Th2 cytokines, we observed no associations between PUFA status and any chemokine or angiogenesis marker.

This study has several strengths. This large cohort had high fish consumption, with concurrent high intake of n‐3 PUFA and relatively high MeHg exposure (10 times higher than in the USA);^74^ therefore, any possible associations with these factors should have been detected in this study. Robust physiologic measures of both MeHg exposure and PUFA status were used, and the cohort size permitted investigation of interactions between maternal MeHg exposure and PUFA status. The study also has a number of limitations. We did not speciate Hg, and although we believe that within the Seychelles population, some 80% of blood THg is MeHg, this ratio may differ in other populations with different exposures and may also be altered during gestation.^75^ The study included only normal pregnancies and all maternal blood samples were taken at 28 weeks’ gestation, so we could not examine MeHg and PUFA exposure across all trimesters. When comparing associations between MeHg and inflammatory markers there is an inherent unreliability in their categorization as pro‐ and anti‐inflammatory which challenges their interpretation.^76^ Inflammatory marker concentrations may also be impacted by time of day, acute stress, possibility of infection, fitness level, and feeding state of the participants, as well as the length of storage time of the blood samples.^77^ The pro‐ and anti‐inflammatory responses of a particular marker may also vary under different activating signals, cell targets, oxidative stress and temporal conditions. The inclusion of ex vivo functional analyses of immune response would have enhanced the understanding and impact of these results. Restrictions, however, in study design and laboratory capabilities prevented this type of analysis in the field. Additionally, the study was cross‐sectional. These and other factors make us cautious in interpreting the data.

## 5 CONCLUSIONS

In this exploratory study, we found associations of maternal MeHg with markers of maternal immune function at 28 weeks’ gestation. Additionally, the n‐3 and n‐6 PUFA were found to have associations, which might indicate suppressive and stimulatory immune responses, respectively. The associations with PUFA were largely independent of MeHg, albeit a significant interaction between MeHg and the n‐6:n‐3 ratio on the Th1:Th2 ratio suggests that the n‐3 PUFA could potentially mitigate the immunosuppressive associations of MeHg observed. Overall, the associations were of small magnitude and of unclear significance given normal immune changes during pregnancy. Further research on these associations is indicated.
